# Dynamic Mechanical Compression of Chondrocytes for Tissue Engineering: A Critical Review

**DOI:** 10.3389/fbioe.2017.00076

**Published:** 2017-12-11

**Authors:** Devon E. Anderson, Brian Johnstone

**Affiliations:** ^1^Department of Orthopaedics and Rehabilitation, Oregon Health & Science University, Portland, OR, United States

**Keywords:** chondrocyte, chondrogenesis, dynamic compression, dynamic loading, bioreactor, tissue engineering

## Abstract

Articular cartilage functions to transmit and translate loads. In a classical structure–function relationship, the tissue resides in a dynamic mechanical environment that drives the formation of a highly organized tissue architecture suited to its biomechanical role. The dynamic mechanical environment includes multiaxial compressive and shear strains as well as hydrostatic and osmotic pressures. As the mechanical environment is known to modulate cell fate and influence tissue development toward a defined architecture *in situ*, dynamic mechanical loading has been hypothesized to induce the structure–function relationship during attempts at *in vitro* regeneration of articular cartilage. Researchers have designed increasingly sophisticated bioreactors with dynamic mechanical regimes, but the response of chondrocytes to dynamic compression and shear loading remains poorly characterized due to wide variation in study design, system variables, and outcome measurements. We assessed the literature pertaining to the use of dynamic compressive bioreactors for *in vitro* generation of cartilaginous tissue from primary and expanded chondrocytes. We used specific search terms to identify relevant publications from the PubMed database and manually sorted the data. It was very challenging to find consensus between studies because of species, age, cell source, and culture differences, coupled with the many loading regimes and the types of analyses used. Early studies that evaluated the response of primary bovine chondrocytes within hydrogels, and that employed dynamic single-axis compression with physiologic loading parameters, reported consistently favorable responses at the tissue level, with upregulation of biochemical synthesis and biomechanical properties. However, they rarely assessed the cellular response with gene expression or mechanotransduction pathway analyses. Later studies that employed increasingly sophisticated biomaterial-based systems, cells derived from different species, and complex loading regimes, did not necessarily corroborate prior positive results. These studies report positive results with respect to very specific conditions for cellular responses to dynamic load but fail to consistently achieve significant positive changes in relevant tissue engineering parameters, particularly collagen content and stiffness. There is a need for standardized methods and analyses of dynamic mechanical loading systems to guide the field of tissue engineering toward building cartilaginous implants that meet the goal of regenerating articular cartilage.

## Introduction

Articular cartilage resides in a complex and dynamic mechanical environment *in vivo*, characterized by compressive and shear stresses along multiple axes and hydrostatic and osmotic pressures throughout (Mansour, 2003; Mow and Huiskes, 2005). The biomechanical environment, in part, governs the development and maturation of the native structural architecture through a classical structure–function relationship (Williams et al., 2008). The heterogeneous and anisotropic extracellular matrix of mature tissue exhibits complex biomechanical properties, including biphasic viscoelasticity and strain stiffening behavior, and the tissue is avascular to accommodate dynamic loading (Mow and Huiskes, 2005). Without a blood supply, articular cartilage lacks the ability for intrinsic repair following disruption of tissue structure and alterations in the mechanical environment during injury. Cartilage injuries range from acute focal tears to chronic degeneration and are a significant cause of disability worldwide (Woolf and Pfleger, 2003; McCormick et al., 2014). To date, tissue-engineered cartilage intended for repair does not exhibit the structural or biomechanical properties of native tissue, and biomaterial-based tissues are governed in part by the mechanical properties inherent to the exogenous scaffold. A mismatch in mechanical properties between a repair tissue and the surrounding native tissue following surgical intervention will necessarily contribute to a continued disruption in joint biomechanics and therapeutic failure. Thus, a successful tissue-engineered construct needs not only to repair the tissue defect but also to integrate into the native tissue to restore joint mechanics to a preinjury state. Bioreactors that recreate one or more components of the mechanical environment of native cartilage have emerged as a primary laboratory tool in tissue engineering; directing tissue development through mechanical stimulation, with the goal of providing the construct with native tissue properties.

**Figure 1:**
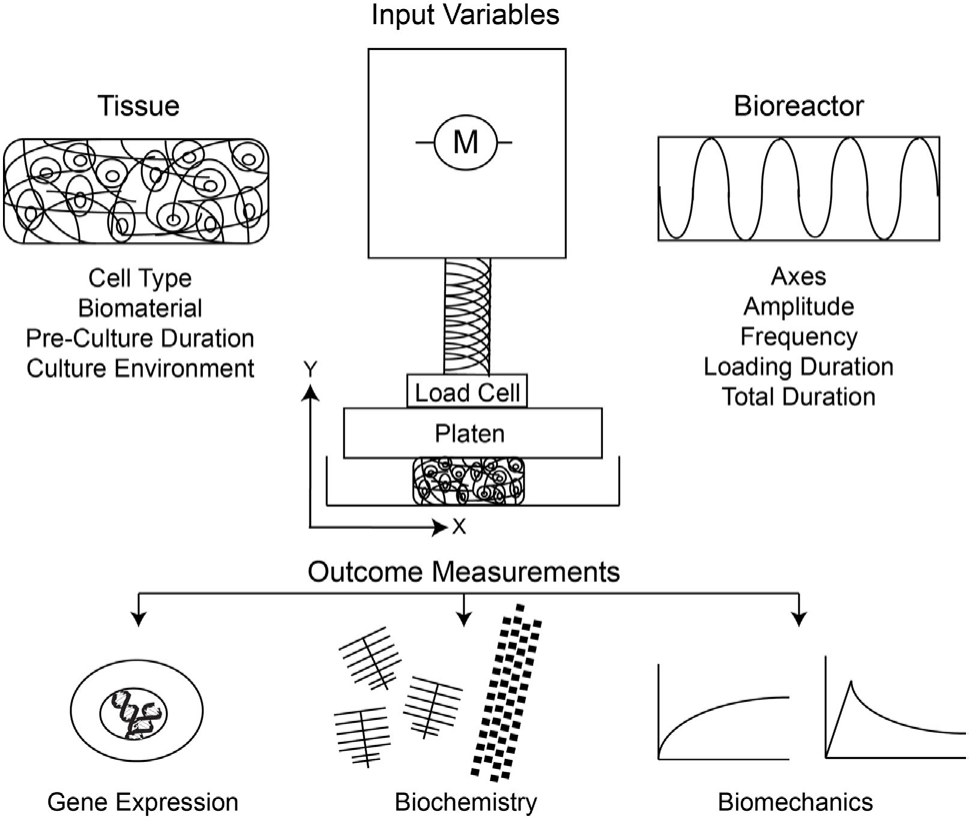
Schematic of the input variables and outcome measurements under consideration in study design, and interpretation thereof, for dynamic compression of chondrocytes.

In the previous century, many groups developed dynamic single-axis compression or multiaxis compression and shear bioreactors to monitor the metabolic and biochemical responses of chondrocytes within native tissue explants to various loading regimes. A major finding was that static compression inhibits but dynamic compression promotes biochemical anabolism (Grodzinsky et al., 2000). Thereafter, investigators sought to characterize the response of biomaterial-seeded chondrocytes to similar loading regimes. Testing the hypotheses that the mechanical environment drives cell differentiation and tissue development *in vitro*, the field of cartilage tissue engineering experienced a rapid expansion of bioreactor-based methods. As research groups independently developed systems to investigate the response of chondrocytes to mechanical stimulation, there was little opportunity for standardization. As a result, the field contains a large body of literature that is varied with respect to both input parameters and outcomes (Figure 1). Input variables, for instance, include those for cells (source, number, state and differentiation), biomaterials (type, fabrication, and preloading culture conditions), bioreactor (type and axes), and loading regime (frequency, amplitude and duration). Furthermore, there is a wide variation in the outcomes measured following bioreactor stimulation that make comparisons more challenging. The most consistently measured outcome parameters are gene expression of various matrix proteins, with protein expression of proteoglycans [actually generally inferred from measuring glycosaminoglycan (GAG) sugar content] and/or collagens often, but not always, included and the bulk mechanical properties frequently, but again not always, included. A limited number of groups have further investigated mechanistic pathways involved in mechanotransduction of loaded chondrocytes. Without standardized loading protocols, the results from these complex and multistep tissue-engineering methods widely differ among individual groups, and the true effect of mechanical loading on chondrocyte-based tissue development remains unclear.

In this review, we sought to summarize the literature regarding dynamic loading of chondrocytes for tissue engineering applications to identify trends based on both input variables and resultant outcomes. Compilation of the literature provides a basis to critically evaluate a wide range of experimental systems relative to one another, as each has been previously reported in isolation. We sought to identify system variables across studies that generate positive or negative results with respect to cartilage tissue engineering, from chondrogenesis measured by gene expression to tissue development measured by biochemical and biomechanical analyses. This thorough review of the expansive literature regarding dynamic compression of chondrocytes illuminates trends that can inform future experimental design for the ultimate goal of engineering tissue with the articular cartilage phenotype.

## Methods

We performed a literature search of the MEDLINE PubMed database for all English language peer-reviewed articles published between January 1, 1995 and December 31, 2016 and that were indexed according to the following search terms in title and/or abstract: (chondrocyte OR chondroprogenitor OR chondrogenesis) AND (dynamic compression OR dynamic loading OR bioreactor OR compression bioreactor OR dynamic compression bioreactor). These searches yielded a total of 1,799 results; however, many individual publications were repetitive with alternative search terms. We then manually assessed the manuscripts to find those in which articular chondrocytes or articular cartilage-derived progenitor cells were used in experiments and dynamic compression was included as at least one element of the loading regime. This resulted in a final list of 63 manuscripts, which we then critically evaluated for their major findings and compared them to each other, noting the many differences in input and output variables used. The results are comprehensively detailed in the tables that accompany this review.

Dynamic loading parameters including frequency, amplitude, duration, and timing clearly influence biochemical and biomechanical outcomes in these studies; however, comparisons between studies should be performed cautiously as each study employs a unique cell source and culture conditions. It is also worth noting that less than half of the compiled studies report sample sizes. In compiling the review, we specifically noted the lack of discussion of species differences by authors when comparing their results with the work of others. Thus, we used this parameter as part of the structural separation of the types of studies we found to emphasize its importance as a variable in the results obtained.

## Results

### Non-Human Large Mammal Chondrocytes in Hydrogel Substrates

#### Effect of Loading Duration

The earliest studies to define the role of dynamic mechanical stimulation of chondrocytes within a tissue engineering system, as opposed to explant culture, used primary bovine chondrocytes seeded into hydrogel-based substrates and investigated the cellular biochemical response, using assays to quantify changes in proteoglycan, collagen, and total protein content. These studies showed that chondrocytes significantly increased proteoglycan synthesis but variably regulated collagen synthesis in response to short-duration dynamic loading at various frequencies, with a majority of positive results at 1 Hz (Buschmann et al., 1995; Lee and Bader, 1997). Using primary bovine chondrocytes seeded at low cell density within agarose hydrogels, Mauck et al. began to define the roles of tissue engineering system parameters, including hydrogel substrate, cell seeding density, growth factor co-stimulation, and serum concentration, finding significant increases in proteoglycan and collagen production, and tissue stiffness with dynamic loading in comparison with free-swelling controls (Mauck et al., 2000, 2003a,b). Importantly, positive results from these studies only arose with greater than 45 total loading hours and appeared to be duration dependent. A time-dependent increase in proteoglycan production, but not overall protein synthesis, was also reported with substantially fewer total loading hours by Chowdhury et al. (2003), with maximal proteoglycan production following an intermittent loading regime of two times 12 h on, 12 h off. Kisiday et al. (2004) similarly reported superior results for an intermittent loading regime, such that alternating days of intermittent dynamic stimulation with days of free-swelling culture drove significant increases in GAG production and accumulation as well as increases in equilibrium and dynamic moduli when compared with free-swelling controls. Another study found that 3 or 6 h of continuous daily loading was superior to a 1 h on/1 h off for three cycles per day protocol in terms of resultant mechanical properties at day 28 (Ng et al., 2009). It can be hypothesized that intermittent loading is superior because the rest period allows the cells to respond to the stimulus with both the production of extracellular matrix molecules and the integration of these molecules into the existing matrix whereas continuous loading drives the newly synthesized molecules out of the tissue through mass transport.

#### Effect of Preload Culture and Timing of Load Initiation

While increased total loading duration has a positive effect on proteoglycan synthesis and accumulation in hydrogel-based systems, the timing of initiation of load application relative to total duration in culture appears to correlate with the resultant hydrogel compressive mechanical properties. Lima et al. showed that application of load immediately following cell seeding caused a significant decrease in GAGs, collagen, Young’s compressive modulus, and dynamic modulus after 32 and 56 days of intermittent loading when compared with free-swelling controls. When application of initial load was delayed by 14 days, there was no difference in GAG or collagen content but there was a significant increase in both moduli in comparison with free-swelling controls (Lima et al., 2007). These results, however, were only partially corroborated by a follow-up study from the same group such that minimal differences existed when the delayed and immediate loading regimes were directly compared (Bian et al., 2010). At the gene level, Nicodemus et al. reported that intermittent loading regimes significantly upregulated both anabolic (*COL2A1* and *ACAN*) and catabolic (*MMP1, MMP3*, and *MMP13*) genes with increased loading duration relative to both preload gene expression and to continuously loaded hydrogels. For the same intermittent regime, investigation of delayed versus immediate loading was performed in a cross-over study with 7 days in free-swelling or dynamic loading with a subsequent switch, and both anabolic and catabolic genes increased under dynamic loading regardless of timing without significant differences in the total GAG content (Nicodemus and Bryant, 2010). Delayed loading likely provides the cells with time to build a preliminary pericellular matrix under chemical stimuli before dynamic loading. The matrix elaborated during a period of delayed stimulation may be necessary for effective mechanotransduction and resultant tissue stiffening in the dynamic mechanical environment.

#### Effect of Cell Source

Bovine and other non-human large mammal-derived chondrocytes are a readily available cell source for laboratory-based tissue engineering protocol development; however, results from experiments utilizing these easily harvested cells appear highly dependent on co-variables of donor age, *in situ* location, and *in vitro* expansion. In comparison with adult-derived chondrocytes, juvenile and neonatal-derived bovine cells have a higher capacity to produce cartilaginous extracellular matrix components at both the gene and protein level (Liu et al., 2013). This effect was noted in a study defining the differences in metabolic response of chondrocytes to dynamic loading based on donor age and location within the joint. Only primary chondrocytes derived from low load-bearing areas of 5-month-old donors significantly increased proteoglycan and protein production with dynamic load in comparison with static load; whereas, cells derived from high load-bearing areas, and neonatal- and adult-derived chondrocytes were unresponsive to the same dynamic loading regime (Wiseman et al., 2003). Farnsworth et al. (2013) similarly showed that while adult chondrocytes that were dynamically loaded at 1 Hz and 5% compression significantly increased total GAGs by 88% at day 14, juvenile chondrocytes loaded at 0.3 Hz and 5% compression significantly increases total GAGs by 220% at day 7 and 280% at day 14, both relative to respective free-swelling controls. Shelton et al. (2003) investigated the differences between deep and superficial chondrocytes, and found that while superficial zone chondrocytes upregulated total protein production under dynamic load at all tested frequencies (0.3, 1, and 3 Hz), only deep zone chondrocytes significantly increased proteoglycan synthesis and likely account for the majority of the response of full-depth chondrocytes to dynamic loading at 1 Hz. These few studies demonstrate a simple principle that not all bovine chondrocytes are equivalent.

#### Effect of Cell Expansion

Primary chondrocytes from large mammals are readily available in quantities sufficient for scalable tissue engineering experiments. Monolayer expansion of any chondrocyte, however, induces modulation of the articular chondrocyte phenotype in a process known as dedifferentiation, which is characterized by a shift from expression of type II to type I collagen at both the gene and protein level (Goessler et al., 2004), loss of gene expression for aggrecan and lubricin (Darling and Athanasiou, 2005), changes to the integrin profile on the cell surface (Goessler et al., 2006a), and differential expression of metabolic mediators and growth factors that regulate extracellular matrix homeostasis (Goessler et al., 2005, 2006b). Though the mechanism for phenotypic modulation is not entirely known, the presence of serum in cell propagation and a two-dimensional growth environment facilitate these changes (Malpeli et al., 2004). A single study investigated the effect of monolayer expansion on the response of bovine chondrocytes to dynamic loading in agarose hydrogels and found that cells passaged once exhibited significantly greater sulfate and thymidine incorporation into matrix components than unstrained controls, however, primary chondrocytes actually significantly decreased sulfate incorporation when loaded compared with free-swelling controls. Chondrocytes expanded beyond two passages significantly decreased both sulfate and thymidine incorporation under dynamic load in comparison with unstrained controls. Cells derived from the same batches but cultured in alginate beads demonstrated the phenotypic modulation from type II to type I collagen with increased passage (Wiseman et al., 2004). Significant differences for results generated with primary versus expanded cells may inhibit translation of the majority of results generated with primary bovine chondrocytes to a human model that will likely necessitate *in vitro* expansion for autologous tissue restoration.

#### Effect of Hydrogel Substrate

Similar to cell factors, hydrogel substrate alone mediates cell response to dynamic loading. When cultured in a fibrin-based hydrogel, bovine chondrocytes responded to dynamic compression in a manner opposite to the previously described agarose-based systems with a significant decrease in GAGs, total collagen, and dynamic stiffness in comparison with free-swelling or statically compressed controls (Hunter et al., 2004). The dramatic differences in chondrocyte response to dynamic load between hydrogel substrates highlights the importance of the cell-matrix interaction, primarily mediated by integrins; however, only agarose and alginate hydrogels have been directly compared in a single study (Mauck et al., 2000). Not all hydrogels are equivalent, and results of these studies must be carefully interpreted to account for differences in substrate.

#### Effect of Soluble Factors

Hydrogel systems have been used to evaluate the influence of co-variables to the dynamic loading culture system, including serum, soluble growth factors, vitamins, and small molecule inhibitors to guide chondrogenic tissue development and evaluate mechanotransduction mechanisms in the dynamic mechanical environment. Mauck et al. (2003a) showed addition of either TGF-β1 and IGF-1 to 10% FBS-supplemented basal media significantly enhanced the response of chondrocytes to dynamic loading with respect to aggregate equilibrium modulus, GAG content, and collagen content, especially with longer duration culture. A study that evaluated the temporal effect of TGF-β supplementation found that a preculture period in TGF-β3 for 14 days before the onset of loading followed by dynamic loading without TGF-β3 primed the constructs for superior mechanical and biochemical properties at 42 days compared with free-swelling controls or with immediate loading and TGF-β3 removal at day 14 (Lima et al., 2007). When cells were seeded at high density of 60 × 10^6^ and loaded in dynamic compression, increasing the FBS concentration from 10 to 20% increased both the Young’s compressive and dynamic moduli but had no effect on collagen or GAG content. This effect was not seen for lower cell seeding density at 10 × 10^6^, which highlights the importance of nutrient demands of cells and transport of those nutrients through tissues in the dynamic environment (Mauck et al., 2003b). Another study using high cell density hydrogels at 60 × 10^6^ showed that addition of ITS and increasing concentration of FBS from 0 to 20% significantly enhanced the biochemical and biomechanical properties of free-swelling constructs in the absence of dynamic load, with the greatest benefit at 2% FBS with ITS supplementation (Kelly et al., 2008). When these constructs were cultured in a dynamic loading environment, the addition of supplements for the entire loading duration was actually detrimental to resultant mechanical properties at days 28 and 42 when compared with free-swelling controls. Priming the constructs in 20% FBS for the first week before transfer to respective FBS concentration plus ITS, rescued the mechanical properties for a significant improvement compared with free-swelling controls (Kelly et al., 2008). These four studies from a single laboratory highlight the importance of media formulation on the tissue level responses to dynamic loading, specifically mechanical properties. Tissues generated in chemically defined media with TGF-β benefit from delayed loading while those cultured in the presence of serum benefit from immediate loading following cell seeding. These differences in biomechanical outcomes may be a result of differences in the biochemical makeup of the extracellular matrix, which was characterized by total proteoglycan and collagen content without a differential analysis into specific types of collagen or other matrix molecules. Increases in tissue stiffness without detectable differences in the bulk collagen or proteoglycan content in some of these studies may represent unmeasured differences in extracellular matrix organization such as collagen type, cross-linking, or organization, which are known to independently modulate overall tissue biomechanical properties in native tissue (Gannon et al., 2015). In consideration of collagen synthesis, Omata et al. (2012) found that increasing the concentration of ascorbic acid in culture media significantly increased the tangent modulus and the pericellular matrix formation compared with free-swelling controls, revealing the importance of ascorbic acid concentration for adequate collagen synthesis.

Adding nutrients to media does not necessarily mean that the cells will have access to them since delivery of nutrients into a thick hydrogel filled with dense extracellular matrix may be limited by diffusion and a consequential nutrient gradient. Dynamic loading inherently increases bulk flow and perfusion through neocartilage tissues, but the addition of nutrient channels to hydrogels has not been shown to further enhance the GAG or collagen profile of tissues beyond that of dynamic loading of solid hydrogels (Mesallati et al., 2013). While bulk flow mediates nutrient delivery in the dynamic system, the tissue is also under the influence of osmotic loading through fixed charge density of the extracellular matrix, and Kelly et al. (2013) showed that dynamic loading in hypotonic salt solution likely removed salts that shield the fixed charges within the tissue and significantly increased the measured mechanical properties, potentially giving a more accurate and promising readout for tissue stiffness. Nutrient availability is relatively easily modeled in a hydrogel system; however, as tissue engineering systems become more complex in both scaffold-based and scaffold-free extracellular matrix tissues, these principles may not hold.

Overall, agarose hydrogels have proven a relatively universal substrate with which to investigate the response of chondrocytes to dynamic compressive loading. This system has allowed the field to systematically investigate: variables of dynamic loading regimes including frequency, strain, duration and timing; differences among cells from varied sources and culture conditions; and additive effects of soluble factors and delivery thereof. These results, which are summarized in Table 1, provide us with very specific parameters by which chondrocytes seeded in hydrogels respond to dynamic compression. We must realize, however, that positive results published in these studies highlight nuances discovered within narrow investigations. For instance, many positive results did not show up until extended culture duration with intermittent loading. Negative data or lack of changes seen in other studies may have simply been the result of too short of study duration to identify positive changes. As seen in the following sections, direct comparison of studies becomes ever more difficult with increasing complexity of tissue engineering and bioreactor systems.

**Table 1:** Compilation of studies that investigated the response of non-human, large mammal-derived chondrocytes seeded into hydrogels and subjected to uniaxial dynamic compressive loading.

Reference	Study design/investigation	Cell source (*n*), hydrogel, and preculture duration	Loading parameters	Results [PG = proteoglycans, Eeq = equilibrium, Edyn = dynamic, Ey = Young’s, and H(A) = aggregate]
Buschmann et al. (1995)	Cellular metabolic response to single dynamic loading period	Neonatal primary bovine chondrocytes in 2 or 3% agarose precultured 3, 13, or 24 days	Frequency: 0.001, 0.01, 0.1, and 1 Hz Amplitude: 1% Duration: 10 h continuous Max duration: 10 h	RNA: not assessed PG: ↑ all frequencies at days 5 and 23 Collagen: ↑ all frequencies at days 5 and 23 Biomechanics: not assessed

Lee and Bader (1997)	Effect of loading frequency	18-month-old primary bovine chondrocytes in 3% agarose precultured for 16 h	Frequency: 0.1, 1, and 3 Hz Amplitude: 15% Duration: 48 h continuous Max duration: 48 h	RNA: not assessed PG: ↑ 1 Hz, ↓ 0.3 Hz, and ↔ 3 Hz Collagen: ↓ all conditions Biomechanics: not assessed

Mauck et al. (2000)	Agarose versus alginate hydrogels and subsequent dynamic loading of agarose hydrogels	3- to 5-month-old primary bovine chondrocytes in 1–5% alginate without preculture	Frequency: 1 Hz Amplitude: 10% Duration: 3× 1 h on/1 h off, 5 days/week for 28 days Max duration: 60 h	RNA: not assessed PG: ↑ on day 21 Collagen: ↑ on day 21 Biomechanics: ↑ Eeq and peak stress on days 21–28

Mauck et al. (2003a)	Effect of TGF-β, IGF 1, and load	2- to 12-day-old primary bovine chondrocytes (*n* = 3) in 2% agarose precultured for 0 or 22 days	Frequency: 1 Hz Amplitude: 10% Duration: 3× 1 h on/1 h off, 5 days/week for 35 days Max duration: 75 h	RNA: not assessed PG: ↑ on day 28–35 Collagen: ↑ on day 35 Biomechanics: ↑ H(A) on day 35, increase with TGF-β

Mauck et al. (2003b)	Cell seeding density in agarose and effect of increased FBS concentration	4- to 6-month-old primary bovine (*n* = 5) in 2% agarose without preculture	Frequency: 1 Hz Amplitude: 10% Duration: 3× 1 h on/1 h off, 5 days/week for 56 days Max duration: 105 h	RNA: not assessed PG: ↑ low cell density on day 42 Collagen: ↑ low cell density on days 28–56 Biomechanics: ↑ Eeq and Edyn, α cell seeding density, and % FBS

Shelton et al. (2003)	Effect of dynamic loading frequency on constructs with full-depth, superficial, or deep chondrocytes	18-month-old primary bovine chondrocytes in 3% agarose without preculture	Frequency: 0.1, 1, and 3 Hz Amplitude: 15% Duration: 48 h continuous Max duration: 48 h	RNA: not assessed PG: full thickness or deep cells: ↓ 0.3 Hz, ↑ 1 Hz, and ↔ 3 Hz; superficial cells: ↓ 0.3 Hz, ↔ 1 Hz, and ↓ 3 Hz Collagen: not assessed Biomechanics: not assessed

Wiseman et al. (2003)	Effect of cell maturity (donor age) on response to dynamic loading and the production of NO as a readout for degenerative processes	Fetal through adult primary equine chondrocytes in 4% agarose without preculture	Frequency: 1 Hz Amplitude: 15% Duration: 24 h continuous Max duration: 24 h	RNA: not assessed PG: ↑ 5-month-old chondrocytes from low-load region, ↔ any other condition Collagen: not assessed Biomechanics: not assessed

Chowdhury et al. (2003)	Effect of continuous versus intermittent dynamic loading on cell metabolism	18-month-old primary bovine chondrocytes in 2% agarose precultured for 1 day	Frequency: 1 Hz Amplitude: 15% Duration: 24 h on/24 h off or 1.5, 3, 6, and 12 h continuous Max duration: 24 h	RNA: not assessed PG: ↑ all conditions Collagen: not assessed Biomechanics: not assessed

Wiseman et al. (2004)	Effect of cell passage on response to loading	Adult primary or expanded bovine chondrocytes in 4% agarose without preculture	Frequency: 1 Hz Amplitude: 15% Duration: 24 h continuous Max duration: 24 h	RNA: not assessed PG: ↓ p0 cells, ↑ p1 and p2 cells, ↔ p3 and p4 (p = passage #) Collagen: not assessed Biomechanics: not assessed

Kisiday et al. (2004)	Effect of varied intermittent loading regimes	Neonatal primary bovine chondrocytes in 0.2 or 0.4% agarose with self-assembling peptides precultured for 0 or 22 days	Frequency: 1 Hz Amplitude: 2.5% Duration: 30 min on/30 min off, 1 h on/1 h off, or 3, 5, 7 h/day for 3, 5, 11, or 39 days Max duration: 150 h	RNA: not assessed PG: ↑ intermittent loading Collagen: not assessed Biomechanics: ↑ Eeq and Edyn for intermittent loading

Hung et al. (2004)	Summary of data regarding loading regimes, growth factors, and cell seeding density	4- to 6-month-old primary bovine chondrocytes (*n* = 3–5) in 2% agarose without preculture	Frequency: 1 Hz Amplitude: 10% Duration: 3× 1 h on/1 h off, 5 days/week for 3, 28, or 56 days Max duration: 105 h	RNA: ↑ ACAN at 3 days only PG: not assessed Collagen: not assessed Biomechanics: ↑ H(A) on days 21–28, ↑ Ey on days 28–56, ↑ Edyn on day 56

Hunter et al. (2004)	Static versus dynamic compression in fibrin-based gel biomaterial	2- to 3-week-old previously frozen primary bovine chondrocytes in fibrin gel precultured for 3 days	Frequency: 0.1, 1 Hz Amplitude: 4% Duration: 10 or 20 days continuous Max duration: 480 h	RNA: not assessed PG: ↓ for 0.1 and 1 Hz at days 10 and 20 Collagen: ↓ for 0.1 and 1 Hz at days 10 and 20 Biomechanics: ↓ Edyn for 0.1 and 1 Hz at days 10 and 20

Mouw et al. (2007)	Role of voltage-gated K+ and Ca2+ channels, stretch-dependent ion channels, and ATP-dependent Ca2+ channels in response to loading	2- to 3-week-old primary bovine chondrocytes in 2% agarose precultured for 1 day	Frequency: 1 Hz Amplitude: 3% Duration: 20 h continuous Max duration: 20 h	RNA: not assessed PG: ↑ dynamic load, decreased with blockage voltage-gated Ca++ channels Collagen: not assessed Biomechanics: not assessed

Mauck et al. (2007)	Effect of loading frequency and duration with gene transcription reporter promoter constructs	3- to 6-month-old primary bovine chondrocytes in 2% agarose precultured for 3 days	Frequency: 0.33, 1, and 3 Hz Amplitude: 10% Duration: 1 or 3 h continuous Max duration: 3 h	RNA: ↑ ACAN and ↓ COL2A1 promotor activity PG: not assessed Collagen: not assessed Biomechanics: not assessed

Lima et al. (2007)	Temporal effect of TGF-β and dynamic loading	4- to 6-month-old primary bovine chondrocytes (*n* = 3–5) in 2% agarose precultured for 0 or 14 days	Frequency: 1 Hz Amplitude: 10% Duration: 3 h/day, 5 days/week for 42 or 56 days Max duration: 105 h	RNA: not assessed PG: ↔ preculture, ↓ loaded from day 0 Collagen: ↔ preculture, ↓ loaded from day 0 Biomechanics: ↑ Ey and Edyn with preculture, ↓ Ey and Edyn when loaded from day 0

Kelly et al. (2008)	Effect of FBS and ITS in mechanical environment	3- to 4-month-old primary bovine chondrocytes (*n* = 4–8 pooled) in 2% agarose without preculture	Frequency: 1 Hz Amplitude: 10% Duration: 3 h/day for 28 or 42 days Max duration: 126 h	RNA: not assessed PG: ↔ 0% FBS/ITS, ↑ 0.2% FBS/ITS, and 20% FBS groups, Collagen: ↔ any group Biomechanics: ↔ Ey for 0% FBS/ITS or 20% FBS, ↑ Ey for 0.2% FBS/ITS and 2% FBS/ITS groups

Ng et al. (2009)	Effect of removal of TGF-β from system	3- to 4-month-old primary bovine chondrocytes (*n* = 5) in 2% agarose without preculture	Frequency: 1 Hz Amplitude: 10% Duration: 3× 1 h on/1 h off or 3 or 6 h continuous for 28 days Max duration: 168 h	RNA: not assessed PG: ↔ any group Collagen:↑ type II and type IX collagen on quant IHC Biomechanics: ↑ Eeq for all loading regimes, ↑ Edyn only 3 or 6 h continuous

Villanueva et al. (2009)	Effect of increasing concentrations of RGD in PEG hydrogel	1- to 2-year-old primary bovine chondrocytes (*n* = 6) in 10% PEG ± RGD precultured for 1 day	Frequency: 0.3 Hz Amplitude: 15% Duration: 48 h continuous Max duration: 48 h	RNA: not assessed relative to control PG: not assessed relative to control Collagen: not assessed relative to control Biomechanics: ↔ with 48 h loading

Nicodemus and Bryant (2010)	Continuous versus intermittent and immediate versus delayed loading regimes	1- to 3–week-old primary bovine chondrocytes (*n* = 2) in PEG precultured for 1 or 7 days	Frequency: 0.3 Hz Amplitude: 15% Duration: 1 h on/1 h off or continuous for 7 or 15 days Max duration: 180 h	RNA: ↑ ACAN continuous load at day 7, ↔ ACAN and ↑ COL2, MMP1, MMP3, and MMP13 intermittent load at day 7, ↔ COL2, ACAN and ↑ MMP1, MMP3 preculture + intermittent load PG: ↑ continuous load over 7 days, ↔ for intermittent load or preculture followed by continuous or intermittent load Collagen: not assessed Biomechanics: not assessed

Stojkovska et al. (2010)	Validate bioreactor and alginate system, not necessarily tissue engineering	6-month-old expanded bovine chondrocytes in 1.5% alginate microbeads without preculture	Frequency: 0.42 Hz Amplitude: 10% Duration: 1 h on/1 h off for 14 days Max duration: 168 h	RNA: not assessed PG: not assessed Collagen: not assessed Biomechanics: ↔ any group

Bian et al. (2010)	Immediate versus delayed loading and addition of shear	2- to 4-year-old expanded canine chondrocytes in 2% agarose precultured for 0, 14, or 28 days	Frequency: 1 Hz Amplitude: 5% Duration: 3 h/day, 5 days/week for 28 days Max duration: 60 h	RNA: not assessed PG: ↔ any group Collagen: ↔ any group Biomechanics: ↑ Ey continuous load at days 28–56, delayed load at day 56 only. ↑ Edyn for all loading regimes at day 56

Kaupp et al. (2012)	Effect of duration of dynamic loading	Adult primary bovine chondrocytes (*n* = 10–15) in 2% agarose without preculture	Frequency: 1 Hz Amplitude: 10% Duration: 20, 30, or 60 min continuous Max duration: 1 h	RNA: not assessed PG: ↑ 20 or 30 min load Collagen: ↔ any group Biomechanics: not assessed

Omata et al. (2012)	Cumulative effects of vitamin C and mechanical load	Adult primary bovine chondrocytes in 1% agarose precultured for 1 day	Frequency: 1 Hz Amplitude: 15% Duration: 6 h/day for 22 days Max duration: 132 h	RNA: not assessed PG: not assessed Collagen: ↔ any group Biomechanics: ↑ Etan with increasing AA2P

Kelly et al. (2013)	Role of osmotic loading in conditioning tissues for response to dynamic load	Adult primary canine chondrocytes (*n* = 3–5 pooled) in 2% agarose precultured for 14 days	Frequency: 1 Hz Amplitude: 10% Duration: 3 h/day, 5 days/week for 42 or 56 days Max duration: 105 h	RNA: not assessed PG: ↔ any group Collagen: ↑ collagen/DNA Biomechanics: ↑ Ey(steady state) and Ey(max incremental) with dynamic compression only with osmotic load

Farnsworth et al. (2013)	Effect of cell maturity (donor age) on the response to dynamic loading	2- to 3-year-old primary bovine or 1–3 week old primary bovine chondrocytes (*n* = 2–3) in PEG without preculture	Frequency: 0.3 and 1 Hz Amplitude: 5 or 10% Duration: 30 min on/90 min off for 16 h/day for 14 days Max duration: 56 h	RNA: not assessed PG: ↑ adult chondrocytes at 1 Hz/5% at day 14; ↑ juvenile chondrocytes at 0.3 Hz/5% at days 7 and 14 Collagen: adult chondrocytes ↑ type II collagen and pericellular type VI with quant IHC; juvenile chondrocytes ↓ type II and VI Biomechanics: not assessed

Mesallati et al. (2013)	Nutrient flow through microchannels versus solid hydrogels	4-month-old expanded porcine chondrocytes (*n* = 1) in 2% agarose without preculture	Frequency: Hz Amplitude: 10% Duration: 2 h/day, 5 days/week for 21 days Max duration: 30 h	RNA: not assessed PG: ↑ for solid gels but not channeled gels Collagen: ↑ for solid and channeled gels Biomechanics: not assessed

### Non-Human Large Mammal Chondrocytes in Non-Hydrogel Substrates

In the age of regenerative medicine, we are tasked with interpreting results from increasingly complex tissue engineering systems. Over the past two decades, a primary focus of regenerative medicine has been on biomaterial science for the production of scaffolds and substrates upon which to build increasingly sophisticated tissues. Two general categories of biomaterials used for soft tissue engineering include synthetic polymers produced through chemical reactions and natural polymers, either protein- or carbohydrate-based, produced by living organisms.

#### Synthetic Polymer Scaffolds

Synthetic polymers including poly-lactic acid, poly-glycolic acid (PGA), and poly-caprolactone can be manufactured to meet specifications including porosity, bioresorption rate, and mechanical stiffness. These properties, in turn, highly influence cell phenotype and modulate the cellular responses to the mechanical environment, specifically through load distribution and mechanotransduction. Unlike the prototypical agarose hydrogel system described earlier, the large variety of scaffold-based substrates make it harder to gain an understanding of the response to chondrocytes to a dynamic loading regime when using them. Nonetheless, it is worth reviewing the results from these studies in isolation. When neonatal primary bovine chondrocytes were seeded into PGA scaffolds, precultured for 21 days, and continuously loaded at either 0.001 or 0.1 Hz and 10 or 50% compression for 24 h, they significantly increased both total protein and GAG production in comparison with free-swelling and 10% statically loaded controls, with greatest effect seen at 0.1 Hz and 50% compression (Davisson et al., 2002). When Seidel et al. (2004) dynamically and intermittently loaded primary bovine chondrocytes in a PGA scaffold for long durations, they noted a significant decrease in total collagen content and no difference in total GAG or mechanical properties when compared with free-swelling controls; however, the distribution of GAGs and collagen varied between resultant tissues based on proposed differences in bulk interstitial flow between experimental groups. At the cellular level, Xie et al. (2006) showed that lapine chondrocytes seeded in poly(l-lactide-co-ε-caprolactone) significantly upregulated *COL2A1* mRNA when subjected to dynamic load compared with static load, that gene expression is directly correlated to loading duration, and that the proximal region of the *COL2A1* gene promotor mediates transcriptional activity under dynamic compression. When the same group investigated extracellular matrix production under dynamic load in the same system, they found that dynamic compression increased both GAG and collagen production when normalized to cell count; however, they did not compare the groups statistically (Xie et al., 2007). Contrary to their first study, they found no difference for *COL2A1, ACAN*, or *COL1A1* expression after one day of dynamic loading or free-swelling culture, and continuous loading completely abrogated *COL2A1* and *ACAN* expression after 6 days. An intermittent loading regime that introduced resting periods between dynamic loading cycles rescued *COL2A1* and *ACAN* expression (Xie et al., 2007). Another investigation utilizing lapine chondrocytes seeded in polyurethane scaffolds showed that both 20% and 30% strain at 0.1 Hz increases *ACAN* expression up to 12 h (Wang et al., 2008). Encapsulation of chondrocytes in a collagen gel derived from bovine skin before seeding into the scaffold increased *COL1A1* expression and decreased *COL2A1* expression as would likely be expected with a type I collagen gel (Wang et al., 2008). None of these experiments compared the dynamically stimulated groups with an unloaded or statically loaded control. While the biomaterial itself influences cellular response to loading, El-Ayoubi et al. (2011) showed that biomaterial morphology and pore size independently mediate the canine chondrocyte viability under static and dynamic loading regimes but did not investigate gene or matrix level changes.

#### Natural Polymer Scaffolds

The manufacturability and tunable properties of synthetic scaffolds make them attractive substrates for tissue engineering, but scaffolds derived from natural polymers are generally more biocompatible and non-toxic to cells. Under static compression in type II collagen scaffolds, adult expanded canine chondrocytes exhibited a time- and dose-dependent decrease in protein and proteoglycan production consistent with static compression of native tissue explants. In comparison with both static compression and free-swelling controls, dynamic compression upregulated both total protein and proteoglycan production, but the majority of newly synthesized proteins were lost from the scaffold to the media with dynamic stimulation (Lee et al., 2003). Lapine chondrocytes seeded into a chitosan and gelatin scaffold significantly upregulated *COL2A1* and *ACAN* expression after 3 h of dynamic loading in comparison with unloaded controls, but *COL2A1* was then significantly downregulated after 9 h of compression. After 3 weeks of dynamic loading for 6 h per day, chondrocytes had significantly proliferated and produced more GAGs than the unloaded controls, but this study did not investigate collagen protein to correlate to gene expression data (Wang et al., 2009). When this same group added collagen gel and varied concentrations of genipin to induce collagen cross-linking to the chitosan scaffold base, they found that dynamic loading and increasing genipin concentration increased cell proliferation but decreased normalized GAG production (Wang and Tsai, 2013). While natural polymers provide a framework for cells in the mechanical environment, the scaffold is substantially different from the extracellular matrix of native tissue. In order the build a tissue more representative of the native structure, a handful of groups have relied on the cells to build an extracellular matrix *in vitro* through scaffold-free tissue engineering approaches before dynamic mechanical stimulation.

### Non-Human Large Mammal Chondrocytes in Scaffold-Free Systems

In contrast to seeding chondrocytes within synthetic or natural polymers, scaffold-free methods utilize a preculture period in which the cells elaborate their own extracellular matrix as the substrate for later loading studies. It should be noted that even in scaffold-free tissue engineering, biomaterial substrates are often used to guide and/or constrain the tissue during its development period. The Kandel group developed an early method of scaffold-free tissue engineering by seeding juvenile bovine primary chondrocytes at high density onto a calcium phosphate surface and culturing the cells for 4 weeks such that the they produce substantial extracellular matrix before dynamic compressive and/or shear loading (Waldman et al., 2003). Once stimulated with uniaxial dynamic compressive load at 5% strain and 1 Hz for 4,000 cycles every 48 h for 4 weeks, the resultant tissues accumulated significantly more proteoglycans and collagen and exhibited a threefold increase in tissue stiffness than controls in static culture (Waldman et al., 2004). The same group later evaluated the cell level response to short-duration dynamic loading. They reported *MMP3* and *MMP13*—genes coding for matrix metalloproteinases 3 and 13 that degrade type II collagen—were maximally and significantly upregulated 2 h after dynamic compression in comparison with static culture. These genes subsequently declined, and *COL2A1* and *ACAN* were maximally and significantly upregulated 12 h after stimulation. At the protein level, MMP13 was most active 6 h after stimulation as measured by both enzyme activity and fold-change of collagen and proteoglycans released into the media, presumably as a result of MMP cleavage. Despite this, stimulated tissues had significantly greater total synthesis and retention of collagen and proteoglycans 24 h after loading than unloaded controls. These results collectively highlight that tissue remodeling, through both catabolic and anabolic mechanisms, is an important response to dynamic compression (De Croos et al., 2006). Another method to create *de novo* cartilage of cell-derived ECM is to preculture chondrocytes in an alginate hydrogel to provide the cells time to produce matrix molecules. The alginate is then dissolved to leave a suspension of cells with pericellular matrix that can be subsequently seeded to form cartilaginous tissues free of exogenous scaffolding. Hoenig et al. (2011) seeded alginate-released porcine chondrocytes onto a hydroxyapatite mold before dynamic stimulation and reported no differences in quantitative measures of GAGs, type I collagen, or type II collagen regardless of loading amplitude when compared with unloaded controls. Dynamic compression at 5 and 10% strain, however, significantly increased the resultant tissue stiffness, and 20% strain significantly increased Young’s modulus, again all in comparison with unloaded controls. In a similar model, Stoddart et al. (2006) cultured alginate-released bovine chondrocytes in a silicon mold for 14 days before application of dynamic load to create *de novo* tissues from cartilaginous ECM, and found that type II collagen and aggrecan mRNA was significantly upregulated within 1 h of loading relative to unloaded controls, remained upregulated with loading through 3 h, but subsequently declined with 4 h of continuous load. Over 4 days of dynamic stimulation and consistent with aggrecan gene expression, total GAG production was significantly reduced with continuous 24-h loading, significantly increased with intermittent loading regimes equivalent to 2–4 h loading per day, and not changed for a loading regime of 2× 30 min per day. Regardless of intermittent loading regime and relative to unloaded controls, chondrocytes continued to produce significantly more GAGs for an additional 7 days after 4 days of loading, and continuously loaded tissues recovered GAG production from an initial detriment. These results are consistent with those from bovine chondrocytes loaded in agarose hydrogels, such that intermittent regimes are favorable to continuous loading in terms of ECM production. Finally, Tran et al. (2011) investigated the response of neonatal porcine chondrocytes in a self-assembly model of scaffold-free tissue engineering by which cells were seeded into non-adherent agarose wells and again provided time for matrix elaboration before further studies. When these tissues were subjected to concurrent perfusion and dynamic load, total GAG content and equilibrium compressive modulus, but not collagen content, are significantly increased in comparison with tissues maintained in static culture (Tran et al., 2011). These results, however, appear attributable to perfusion alone since they persist in the absence of dynamic loading.

Overall, the response of non-human, large mammal chondrocytes seeded into scaffolds and subjected to dynamic compression modestly favors dynamic compression over static compression and free-swelling controls. These results are summarized in Table 2. The complexity of scaffold-based systems, however, means that the conclusions drawn from more controlled hydrogel-based systems are not fully supported. It is thus fascinating that despite being conducted with mostly young chondrocytes without monolayer preculture (and thus phenotypic modulation), these studies taken together do not definitively show that dynamic compression drives ECM production and biomechanical stiffening, a phenomenon that is accepted as fact in cartilage science. The following sections, summarizing results with chondrocytes and chondroprogenitors of other species, indicate just how different the conclusions are without standardization of input and output parameters.

**Table 2:** Compilation of studies that investigated the response of non-human, large mammal-derived chondrocytes seeded into biomaterial scaffolds and subjected to uniaxial dynamic compressive loading.

Reference	Study design/investigation	Cell source (*n*), scaffold, and preculture duration	Loading parameters	Results [PG = proteoglycans, Eeq = equilibrium, Edyn = dynamic, Ey = Young’s, H(A) = aggregate]
Davisson et al. (2002)	Static versus dynamic compression	Neonatal primary bovine chondrocytes (*n* = 2) in poly-glycolic acid (PGA) scaffold precultured for 21 days	Frequency: 0.001 and 0.1 Hz Amplitude: 5% Duration: 24 h continuous Max duration: 24 h	RNA: not assessed PG: ↑ dependent on static preload and frequency Collagen: ↑ dependent on static preload and frequency Biomechanics: not assessed

Lee et al. (2003)	Static versus dynamic loading regimes in comparison with explants	Adult expanded canine chondrocytes in type II collagen scaffold precultured for 2, 7, 14, or 30 days	Frequency: 0.1 Hz Amplitude: 3% Duration: 24 h continuous Max duration: 24 h	RNA: not assessed PG: ↑ total protein, ↔ proteoglycan Collagen: not assessed Biomechanics: not assessed

Seidel et al. (2004)	Static versus dynamic compression combined with perfusion	Calf primary bovine chondrocytes in PGA scaffold precultured for 30 days	Frequency: 0.3 Hz Amplitude: 5% Duration: 1 h/day Max duration: 37 h	RNA: not assessed PG: ↔ static and dynamic load Collagen: ↓ static and dynamic load Biomechanics: ↔ Eeq static and dynamic load

Waldman et al. (2004)	Study short-term loading regimes to optimize conditions for longer duration loading	6- to 9-month-old primary bovine chondrocytes on calcium phosphate surface precultured for 28 days	Frequency: 1 Hz Amplitude: 5, 10, or 20% Duration: either 400 or 2,000 cycles every 48 h for 7, 14, or 28 days Max duration: 2.8 h	RNA: not assessed PG: ↑ glycosaminoglycan/DNA at day 28 Collagen: ↑ collagen/DNA at day 28 Biomechanics: ↑ Eeq at day 28

De Croos et al. (2006)	Catabolic activity and mitogen-activated protein kinase mechanotransduction in dynamically stimulated tissues	6- to 9-month-old primary bovine chondrocytes (*n* = 2–3) on calcium phosphate surface precultured for 3 days	Frequency: not reported Amplitude: 1.4% Duration: 30 min continuous Max duration: 30 min	RNA: ↑ COL2, ACAN only at 12 h; ↑ MMP3, MMP13 only at 2 h PG: ↑ all conditions Collagen: ↑ all conditions Biomechanics: not assessed

Stoddart et al. (2006)	Loading duration and intermittent loading regimes	10-month-old primary bovine chondrocytes in alginate-released matrix precultured for 14 days	Frequency: 0.276 Hz Amplitude: 0.5 N Duration: varied 30 min to 8 h for 4 days Max duration: 32 h	RNA: ↑ COL2, ACAN only from 1 to 3 h load, ↔ 4 h of continuous load PG: ↑ 2× 30 min or 2× 2 h loading, ↓ continuous loading Collagen: not assessed Biomechanics: not assessed

Xie et al. (2006)	Mechanism of COL2A1 upregulation with dynamic compression	6-week-old expanded lapine chondrocytes in poly(l-lactide-co-ε-caprolactone) (PLCL) sponge precultured for 2 days	Frequency: 0.1 Hz Amplitude: 10% Duration: 24 h continuous Max duration: 24 h	RNA: ↑ COL2 with activity at proximal promoter PG: not assessed Collagen: not assessed Biomechanics: not assessed

Xie et al. (2007)	Role of loading duration and frequency on gene expression	6-week-old expanded lapine chondrocytes in PLCL sponge precultured for 3 days	Frequency: 0.01, 0.05, 0.1, or 0.5 Hz Amplitude: 10% Duration: continuous or intermittent (6 h on/6 h off or 12 h on/12 h off) for 1, 3, or 6 days Max duration: 72 h	RNA: ↔ COL1, COL2, and ACAN for any loading regime. No comparison to control PG: ↑ at days, 3, and 6 of continuous load, no comparison to control Collagen: ↑ at days 1 and 3 of continuous load, no comparison to control Biomechanics: not assessed

Wang et al. (2008)	Role of dynamic compression on chondrogenic gene expression, not comparison to unloaded or static controls	Neonatal expanded lapine chondrocytes in mixed polyurethane and collagen matrix precultured for 1 day	Frequency: 0.1 Hz Amplitude: 20 or 30% Duration: 4, 8, 12, or 24 h continuous Max duration: 24 h	RNA: ↑ ACAN at 30% compression, no comparison to unloaded control PG: not assessed Collagen: not assessed Biomechanics: not assessed

Wang et al. (2009)	Single loading regime outcomes	Neonatal expanded lapine chondrocytes in a 2% chitosan + 2% gelatin matrix precultured for 3 days	Frequency: 0.1 Hz Amplitude: 40% Duration: 6 h/day for 7 or 21 days Max duration: 126 h	RNA: ↑ COL2, ACAN with 3 h load, ↓ COL2 with 9 h load, ↔ COL1 PG: ↑ 3 weeks loading only Collagen: not assessed Biomechanics: not assessed

El-Ayoubi et al. (2011)	Effect of varied biomaterial combinations, not functional tissue	Adult expanded canine chondrocytes in bioprinted PLLA scaffold precultured for 1 day	Frequency: 1 Hz Amplitude: 10% Duration: 1 h on/7 h off continuous cycling for 14 days Max duration: 56 h	RNA: not assessed PG: not assessed Collagen: not assessed Biomechanics: not assessed

Hoenig et al. (2011)	Response of scaffold-free tissues to variable compressive strain amplitudes	4- to 6-month-old expanded porcine chondrocytes (*n* = 5) in alginate-released matrix and on hydroxyapatite mold without preculture	Frequency: 1 Hz Amplitude: 5, 10, or 20% Duration: 5 min on/30 min off for total 6 h/day for 14 days Max duration: 84 h	RNA: not assessed PG: ↔ quantitative histology Collagen: ↔ quantitative IHC Biomechanics: ↑ stiffness at 5 and 10% load strain ↑ Ey at 20% load strain

Tran et al. (2011)	Response of scaffold-free tissues to dynamic stimulation and perfusion	Neonatal primary porcine chondrocytes (*n* = 5) in self-assembled matrix precultured for 17 days	Frequency: 1 Hz Amplitude: 0.5 N first week, 10 N second week, and 20 N third week Duration: 4 h/day, 5 days/week for 21 days Max duration: 60 h	RNA: not assessed PG: ↑ perfusion and dynamic load Collagen: ↔ any group Biomechanics: ↑ Eeq and Edyn at final time point

Wang et al. (2013)	Varied concentrations of genipin for collagen cross-linking	Primary lapine chondrocytes in a chitosan/collagen cross-linked matrix precultured for 3 days	Frequency: 0.1 Hz Amplitude: 40% Duration: 30 min/day for 14 days Max duration: 7 h	RNA: not assessed PG: ↓ at final time Collagen: not assessed Biomechanics: not assessed

### Rodent Chondrocytes in Hydrogels and Scaffolds

There are a number of studies that have investigated the response of rodent chondrocytes to dynamic mechanical compression in agarose hydrogels or collagen scaffolds. In addition to isolating articular chondrocytes, several groups have investigated the response of costal-derived chondrocytes, presumably due to the difficulty of isolating sufficient cell numbers from articular cartilage. Over a long-duration intermittent dynamic loading regime, murine costal chondrocytes cultured in 2% agarose gels significantly upregulated type II collagen gene and protein expression with a concomitant increase in the aggregate modulus when compared with unloaded controls (Chokalingam et al., 2009). Consistent with results from bovine chondrocyte studies described earlier, tissue-level responses were only evident with increased total duration in dynamic culture. These similarities between species and chondrocyte source offer some model validity to the agarose hydrogel system. Another study that dynamically compressed expanded rat chondrocytes embedded in agarose hydrogels showed that an increase in total GAG content is a delayed response following continuous loading for 24 h, and GAG production persists with continued free-swelling culture (Tsuang et al., 2008).

As large-scale tissue engineering studies are limited by cell number, the majority of studies utilizing rodent chondrocytes focused primarily on cellular level responses to dynamic mechanical stimulation, specifically gene expression and mechanotransduction pathway analysis, instead of tissue level responses of biochemical and biomechanical properties. Bougalt et al. demonstrated that chondrocytes seeded in agarose gels had phosphorylated ERK1/2 and p38 within 15 min following dynamic compressive stimulation, and these proteins were subsequently dephosphorylated within 60 min. Chondrocytes additionally significantly downregulated *COL2A1* promotor activity with compressive load in comparison with unloaded controls (Bougault et al., 2008). These results are consistent with earlier studies that demonstrated ERK1/2 phosphorylation is mediated by the release of FGF2 from the pericellular matrix on short-duration cyclic loading of porcine cartilage explants and chondrocytes seeded in alginate gels (Vincent et al., 2004, 2007). FGF2 is known to upregulate catabolic enzymes including matrix metalloproteinases 1 and 3 (MMP1, MMP3) and anti-catabolic factors including tissue inhibitor of metalloproteinases 1 (TIMP1) in response to cartilage injury through induction of the mitogen-activated protein kinase pathway (Vincent et al., 2002; Chong et al., 2013). Bougault et al. (2012) later demonstrated with microarray analysis that murine chondrocytes subjected to dynamic loading downregulated 85% of the identified mechanosensitive genes with short-duration stimulation, but downstream TGF-β signaling of smad2 was activated under loading conditions. TGF-β signaling is well known to regulate cell proliferation and chondrogenic differentiation through Sox9 transcriptional control of genes coding for extracellular matrix molecules, primarily type II collagen and aggrecan of articular cartilage. Consistent with this mechanism, primary rat chondrocytes in a collagen I gel were compressed for 60 min per day for 7 days and significantly upregulated *COL2A1* and *ACAN* expression with concomitant decrease in *COL1A1* compared with unloaded controls and longer or shorter daily loading durations (Ando et al., 2009). Addition of exogenous FGF2 or BMP2 independently increased *COL2A1* and *ACAN* mRNA expression; however, each growth factor diminished chondrogenic matrix production when combined with dynamic stimulation, which suggests that proliferation driven by exogenous growth factors can override differentiation induced by mechanical stimulation. Temporally restricted gene expression alone, however, does not necessarily predict tissue-level protein responses to either growth factor or dynamic mechanical stimulation. A combinatorial analysis of the addition of serum to growth media, dynamic compression, or both serum and compression showed that higher concentrations of serum suppress matrix catabolism during tissue formation while dynamic compression increases catabolic activity to potentially rearrange molecules in the resultant tissue (Wu et al., 2013). Unfortunately, experiments testing the overall effect of serum on cell metabolism are unable to distinguish individual contributions of the myriad soluble factors within serum to correlate these results with those testing individual growth factors. Taken together, this set of studies that evaluated the responses of rodent-derived chondrocytes to dynamic stimulation (summarized in Table 3) highlight the importance of tissue remodeling during tissue development and growth in the mechanical environment, but the details of this metabolic regulation under dynamic loading remain vague in our current literature.

**Table 3:** Compilation of studies that investigated the response of rodent-derived chondrocytes subjected to uniaxial dynamic compressive loading.

Reference	Study design/investigation	Cell source (*n*), scaffold, and preculture duration	Loading parameters	Results [PG = proteoglycans, Eeq = equilibrium, Edyn = dynamic, Ey = Young’s, H(A) = aggregate]
Bougault et al. (2008)	Mechanotransduction through ERK and p38 pathways	Embryonic primary murine costal chondrocytes in 2% agarose hydrogel precultured for 7 days	Frequency: 2 s on/1 s off Amplitude: 20 kPa Duration: 30 min continuous Max duration: 30 min	RNA: not assessed PG: not assessed Collagen: not assessed Biomechanics: not assessed

Tsuang et al. (2008)	Effect of dynamic compression on chondrocyte metabolism	Expanded rat chondrocytes in 3% agarose hydrogel without preculture	Frequency: 0.5, 1, 2, or 3 Hz Amplitude: 5, 10, or 15% Duration: 24 h continuous Max duration: 24 h	RNA: not assessed PG: ↑ 10–15% strain and 1 Hz Collagen: not assessed Biomechanics: not assessed

Chokalingam et al. (2009)	Effect of dynamic compression on construct stiffness and collagen expression in col2 reporter mice	Neonatal expanded murine costal chondrocytes (*n* = 6) in 2% agarose hydrogel precultured for 2 days	Frequency: 1 Hz Amplitude: 10% Duration: 3× 1 h on/1 h off, 5 days/week for 7, 14, 21, or 28 days Max duration: 60 h	RNA: ↑ COL2 for all time points with max at day 14 PG: not assessed Collagen: ↑ type II collagen content at days 21 and 28 Biomechanics: ↑ H(A) at day 28 only

Ando et al. (2009)	Effect of mechanical loading and growth factors	5-week-old primary rat chondrocytes in type I collagen scaffold without preculture	Frequency: 0.33 Hz Amplitude: 5% Duration: 10, 60, or 120 min/day for 7 days Max duration: 14 h	RNA: ↑ ACAN, COL2 only 60 min/day loading, ↓ COL1 all groups PG: not assessed Collagen: not assessed Biomechanics: not assessed

Bougault et al. (2012)	Early molecular events triggered by dynamic loading including mitogen-activated protein kinase (MAPK) and TGF-β signaling pathways	Embroyonic primary murine costal chondrocytes in 2% agarose hydrogel precultured for 6 days	Frequency: 0.5 Hz Amplitude: 20–40 kPa Duration: continuous for 5, 15, or 30 days Max duration: 720 h	RNA: not assessed for chondro genes: investigated the MAPK, SMAD signaling pathways PG: not assessed Collagen: not assessed Biomechanics: not assessed

Wu et al. (2013)	Effect of serum and dynamic load on spatiotemporal pericellular matrix distribution	Neonatal primary murine costal chondrocytes (*n* = 50 pooled) in 2% agarose hydrogel without preculture	Frequency: 1 Hz Amplitude: 10% Duration: 5 h/day, 7 days/week for 1, 7, 9, 15, or 21 days Max duration: 105 h	RNA: ↑ COL2A1 with load in no serum, ↓ ACAN with load regardless serum, ↔ COL6A1, ↑ COMP with load in serum-free and 10% FBS, ↑ MMP3 with load, ↓ MMP13 with load and no serum or 10% FBS. PG: ↓ all load Collagen: ↓ type II collagen width of distribution from cell with loading Biomechanics: not assessed

### Healthy and Osteoarthritic Human Chondrocytes in Hydrogels, Scaffolds, and Scaffold-Free Systems

Ultimately, the field of articular cartilage tissue engineering seeks to develop translatable therapies to repair focal traumatic defects in adult human populations, yet few groups have investigated the response of adult human chondrocytes to dynamic compressive loading. Of those studies that utilized human chondrocytes, the majority used expanded cell populations from osteoarthritic tissue, which is readily available as discarded tissue from total joint arthroplasty. In contrast to cells isolated from bovine tissue, primary human chondrocyte yields are relatively poor, whether utilizing autologous or allogeneic chondrocytes, and studies that employ human chondrocytes almost always expand cells *in vitro* to achieve sufficient cell numbers for experimentation. A single study to utilize expanded chondrocytes derived exclusively from healthy human donors found high intra-donor variability with regard to GAG production in dynamic compression, such that the cells that showed the greatest response to loading were those that produced abundant GAGs before application of load (Démarteau et al., 2003). This study found no difference in gene expression with application of load. The remainder of studies utilized chondrocytes derived from osteoarthritic tissue. When gene expression analysis was investigated temporally by collecting RNA at defined time points following the completion of a loading regime, Jeon et al. (2012) reported a significant increase in *ACAN, COL2A1, COL1A1, COL10A1*, and *PRG4* only 2 h after a single 1 h loading period of osteoarthritic chondrocytes seeded in alginate hydrogels, and the response was greater for superficial zone-derived cells than for those derived from the middle zone of diseased tissue. This study also showed that *ACAN* and *PRG4* expression was significantly increased in both superficial and middle zone chondrocytes at 50% strain relative to 5 and 15% strain and after 3 h of loading relative to 1 or 12 h of loading at 15% strain. Relative to unloaded controls, superficial zone-derived chondrocytes, but not middle zone-derived cells, significantly increased GAG content, equilibrium modulus, storage/loss moduli, and type II collagen and aggrecan protein expression when dynamically compressed under the optimized loading regime of 3 h/day at 50% strain and 1 Hz for 2 weeks (Jeon et al., 2012). A follow-up study by the same group showed that both superficial and middle zone-derived chondrocytes that were precultured in hydrogels for 2 weeks before the onset of dynamic loading responded more sensitively to loading than those without preculture and had increased gene expression (*COL2A1, COL1A1, ACAN, FN1, IL1B, IL4*, and *MMP2*) and matrix accumulation (type II and VI collagens, fibronectin, and laminin) when compared with unloaded controls (Jeon et al., 2013). Another study that employed the same commercial bioreactor as that used by Jeon et al. but with an added perfusion system showed modest effects of culturing human chondrocytes under perfusion with non-significant increases in *COL2A1, COL1A1, COL6A*, and *PRG4* gene expression and a decrease in GAG/DNA relative to free-swelling controls (Grogan et al., 2012). Adding a dynamic compression regime of 20% strain, 0.5 Hz, and 1 h/day to perfusion had no further effect on gene expression or ECM production. These results are similar to those presented above for porcine chondrocytes cultured under perfusion and dynamic compression in the bioreactor system, such that increases in GAG content and equilibrium modulus were attributable to perfusion in the absence of compressive loading (Tran et al., 2011). Both of these studies, however, did not investigate alternative loading regimes or optimize the time frame after loading in which to investigate gene expression.

In accordance with other mammalian studies, increased total dynamic compression duration of human osteoarthritic chondrocytes in type I collagen hydrogels significantly increased ECM production (type II collagen via quantitative IHC), gene expression (*COL2A1, ACAN, MMP13*, and *COL1A1*), and stiffness when compared with unloaded controls after 28 days in culture (Nebelung et al., 2012a); these results from 28 days dynamic stimulation were not significant after just 14 days in culture (Nebelung et al., 2012b). Similarly, dynamic compression of human osteoarthritic chondrocytes in a type I collagen gel for 7 days resulted in no significant differences in *SOX9, COL2A1, ACAN*, or various integrin genes, nor a change in GAG/DNA, relative to free-swelling controls, but they did find increased intracellular Sox9 transcription factor and decreased collagen degradation products with loading potentially favoring early activation of anabolism for eventual matrix production (Diao et al., 2017). A more thorough investigation of the metabolome of human osteoarthritic chondrocytes seeded in agarose gels identified hundreds of target metabolites that were responsive to dynamic compression (Zignego et al., 2015). Each metabolome was patient- and age-specific, but consistently showed an increase in metabolites involved in central energy production, such as glycolysis and TCA cycle, and chondroitin sulfate degradation mechanisms. To date relatively few studies have investigated the response of human chondrocytes to dynamic compression (summarized in Table 4), and these studies have almost exclusively utilized chondrocytes derived from diseased tissue. To work toward production of tissue-engineered human articular cartilage for repair of focal defects in otherwise healthy tissue, standardization of cell source and compression protocols will be crucial to derive meaningful results for translation of laboratory studies to clinical application.

**Table 4:** Compilation of studies that investigated the response of human-derived chondrocytes subjected to uniaxial dynamic compressive loading.

Reference	Study design/investigation	Cell source (*n*), scaffold, and preculture duration	Loading parameters	Results [PG = proteoglycans, Eeq = equilibrium, Edyn = dynamic, Ey = Young’s, and H(A) = aggregate]
Démarteau et al. (2003)	Effect of culture duration and donor variability	22- to 47-year-old expanded human chondrocytes from healthy donors (*n* = 4) in PEGT/PBT (55:45) scaffold precultured for 3 or 14 days	Frequency: 0.1 Hz Amplitude: 5% Duration: 2 h on/10 h off for 6 total cycles Max duration: 12 h	RNA: ↔ COL2, COL1, ACAN, VCAN, and SOX9 PG: highly variable b/n donors, trended ↓ Collagen: not assessed Biomechanics: not assessed

Nebelung et al. (2012a)	Effect of dynamic compression on gene expression and mechanical stiffness	67-year-old (mean) primary human chondrocytes from osteoarthritic donors (*n* = 12) in type I collagen hydrogel precultured for 0.5 days	Frequency: 0.3 Hz Amplitude: 10% Duration: 14 days continuous Max duration: 336 h	RNA: ↑ ratio COL2:COL1, ↔ COL2, COL1, ACAN, and MMP13 PG: ↔ Collagen: ↔ Biomechanics: ↓ Eeq relative to baseline, no diff with loading

Nebelung et al. (2012b)	Effect of long-term continuous dynamic compression	67-year-old (mean) primary human chondrocytes from osteoarthritic donors (*n* = 8) in type I collagen hydrogel precultured for 0.5 days	Frequency: 0.3 Hz Amplitude: 10% Duration: 28 days continuous Max duration: 672 h	RNA: ↑ COL2, MMP13, and COL1 after 28 days. ↔ for ACAN PG: not quantified Collagen: ↑ type II on IHC Biomechanics: not significant ↑ Eeq

Grogan et al. (2012)	Combinatorial effect of perfusion and dynamic compression	14- to 55-year-old expanded human chondrocytes from healthy or osteoarthritic donors (*n* = 9) in 2% alginate hydrogel precultured for 1 or 2 days	Frequency: 0.5 Hz Amplitude: 20% Duration: 1 h/day for 7 or 14 days Max duration: 14 h	RNA: ↔ COL2, COL6, COL10, ACAN, PRG4, COL1, MMP3, iNOS, and CCL20 for perfusion or perfusion + load PG: ↓ in glycosaminoglycan (GAG)/DNA for perfusion and load Collagen: not quantified Biomechanics: ↔ stiffness

Jeon et al. (2012)	Zonal chondrocyte differences in response to loading, characterized timeline of gene changes	49- to 78-year-old expanded human chondrocytes from osteoarthritic donors (*n* = 4) in 2% alginate precultured for 14 days	Frequency: 1 Hz Amplitude: varied Duration: 3 h/day for 14 days Max duration: 42 h	RNA: ↑ ACAN, COL2, COL1, COL10, and PRG4 for superficial chondrocytes 2 h post-compression. ↑ ACAN, PRG4, and COL1 at 3 h PG: ↑ retained and total Collagen: ↑ type II *via* quant IHC Biomechanics: ↑ Eeq, E(storage), E(loss)

Jeon et al. (2013)	Differences between zonal chondrocytes in response to loading and preculture duration	Primary or expanded human chondrocytes from osteoarthritic donors in 2% alginate hydrogel precultured for 1 or 14 days	Frequency: 1 Hz Amplitude: 50% Duration: 3 h/day for 14 days Max duration: 42 h	RNA: ↑ COL2, COL1, ACAN, FN1, and HSP2 after 14 days of preculture and 14 days loading. Investigated suite of inflammatory genes PG: not assessed Collagen: ↑ in type II and VI via quantitative IHC Biomechanics: not assessed

Zignego et al. (2015)	Changes in chondrocyte metabolomic profile with loading	50- to 84-year-old expanded human chondrocytes from osteoarthritic donors (*n* = 5) in 4.5% agarose hydrogel without preculture	Frequency: 1.1 Hz Amplitude: 5% Duration: 15 or 30 min continuous Max duration: 30 min	RNA: metabalome analysis only: glycolysis and central energy metabolism ↑ with load PG: metabalome analysis only: chondroitin sulfate degradation pathway ↑ with load Collagen: not assessed Biomechanics: not assessed

Diao et al. (2017)	Regulation of catabolic genes with loading	60- to 80-year-old expanded human chondrocytes from osteoarthritic donors (*n* = 8) in type I collagen microcapsules precultured for 4 days	Frequency: 1 Hz Amplitude: 10% Duration: 3 h/day for 1 or 7 days Max duration: 21 h	RNA: ↔ SOX9, COL2, ACAN, integrins, MMP14, ↓ MMP1, 2, and 13 PG: ↔ GAG/DNA Collagen: not assessed Biomechanics: not assessed

### The Next Dimension: Addition of Shear to Dynamic Loading Regimes

The overall goal of stimulating chondrocytes *in vitro* for tissue regeneration is to provide the cells with mechanical cues representative of those in the native joint environment, and dynamic shear loading is a well-defined component of the native loading regime during articulation. Due to complexity of bioreactor design needed to integrate dynamic compression and shear loading, few groups have investigated the additive effect of dynamic shear to a compressive loading regime. An initial study by Waldman et al. (2003) showed that relative to scaffold-free tissues in static culture, dynamic 5% compressive strain of bovine chondrocytes cultured on a ceramic surface slightly increased proteoglycan and collagen content and equilibrium modulus, and the addition of 2% shear strain to the compressive regime significantly enhanced these tissue-level outcomes. A follow-up study in the same tissue and bioreactor systems reported that tissues subjected to a loading regime of 5% compressive and 5% shear strains for one week produced greater amounts of collagen and proteoglycans relative to both unloaded controls and loading regimes of 2 and 2%, 2 and 5%, and 5 and 2% compressive and shear strains, respectively (Waldman et al., 2007). When subjected to 4 weeks of dynamic stimulation at 5% compressive and 5% shear strains, tissues accumulated 46% more collagen and 54% more proteoglycans compared with unstimulated controls. The increase in ECM accumulation was complemented by a 3-fold increase in compressive modulus and 1.75-fold increase in shear modulus of the stimulated tissues compared with those cultured in the absence of multiaxial loading. While this group previously reported results for tissues subjected to a uniaxial compressive regime, they did not include direct comparison to this group in the multiaxial studies, which limits the ability to directly compare outcomes.

Another group that pioneered use of a multiaxial bioreactor initially compared dynamic compression, dynamic shear, and combinations of compression and uniaxial or multiaxial shear loading regimes for bovine chondrocytes seeded in polyurethane scaffolds (Grad et al., 2006). Compared with free-swelling controls, dynamic compression or uniaxial oscillating rotation of the scaffold (shear) had no effect on the mRNA expression levels of *PRG4, ACAN, COMP, COL1A1, COL2A1, MMP3, MMP13, TIMP1*, or *TIMP3*. The combination of dynamic compression with either unidirectional or multidirectional shear-inducing oscillation, however, resulted in a significant upregulation of several genes, including *PRG4, ACAN, COMP, COL2A1, TIMP3*, and a downregulation of *MMP13*, which again favors anabolism. At the protein level, combined compression and shear led to significantly increased levels of lubricin, COMP, and hyaluronic acid released into the culture media relative to unloaded controls, isolated compression, or isolated shear. These phenotypic changes at the gene and protein level correlate with upregulation of the articular chondrocyte phenotype. Over long-duration loading in the same system, lower oxygen levels further improved the phenotype with increased GAG/DNA content though not to the significance achieved from the effect of lowered oxygen on unloaded controls (Wernike et al., 2007). At the gene level, loading in low oxygen conditions significantly reduced *COL1A1* gene expression to further favor the articular chondrocyte phenotype. Consistent with results from bovine cells cultured in agarose hydrogels under uniaxial compression, longer duration and delayed application of combined dynamic compressive and shear loading of bovine cells in polyurethane scaffolds produced tissues of significantly greater GAG content and favored upregulation of articular chondrocyte genes *COL2, ACAN, COMP*, and *PRG4* (Wang et al., 2013). These cells, however, still highly expressed type I collagen at the gene and protein levels in a polyurethane scaffold regardless of loading regime, and primary chondrocytes responded more favorably than expanded chondrocytes to dynamic loading with respect to GAG production and chondrogenic gene expression. Toward the goal of further modeling the *in situ* cartilage environment for cartilage regeneration *in vitro*, Hilz et al. (2013) added an electromagnetic field as a variable to the dynamic compression and shear loading of bovine chondrocytes in polyurethane scaffolds to mimic a fixed charge density gradient in native tissue. They found that stimulation with load and a 3 mT electromagnetic field produced significantly greater GAG/DNA compared with static control, mechanical stimulus only, and 3 mT field only (Hilz et al., 2013). Continued experimentation utilizing the bioreactor, tissue engineering scaffold system, and cells initially described by Grad et al. has allowed this group to define increasingly specific parameters that favor dynamic loading regimes of combined compressive and shear loading. When a foundational variable was changed by utilizing human-derived cloned articular cartilage progenitor cells instead of primary bovine chondrocytes, this group found a consistent increase in GAG content for loaded tissues relative to unloaded controls, although they did not find significant differences in gene expression (Neumann et al., 2015).

Few other groups have integrated shear into a multiaxial dynamic compressive loading regime. Bian et al. (2010) reported that the addition of shear loading to dynamic compression for tissues derived from expanded canine chondrocytes seeded in agarose hydrogels not only significantly decreased the coefficient of friction but also significantly increased the Young’s compressive modulus relative to unloaded controls and all other dynamic compression regimes. These results, however, only became significant at 56 days in culture. Another study with substantially shorter preculture and loading durations, found no difference in the amount of GAGs produced by tissues under various dynamic shear, compression, and perfusion regimes (Pourmohammadali et al., 2013). Finally, a study that utilized human fetal epiphyseal chondrocytes cultured at varied cell density in either PGA scaffolds or PGA-alginate scaffold-hydrogels sought to characterize differences between preculture in a shaking flask or a perfusion bioreactor before subsequent loading in a dynamic shear and compressive bioreactor (Shahin and Doran, 2011). They found that preculture in a perfusion bioreactor followed by short-duration dynamic stimulation produced the greatest quantity of GAGs and total collagen; however, they did not analyze the individual effects of compression or shear, and they tested many confounding variables in each experiment.

Taken together, studies that added shear to a dynamic compressive loading regime suggest that shear has a significant effect on chondrocyte gene expression, tissue extracellular matrix metabolism, and tissue mechanical properties (Table 5). This conclusion is convincingly shown in studies that introduced shear as a single variable for comparison with dynamic compressive loading regimes.

**Table 5:** Compilation of studies that investigated the response of chondrocytes subjected to multiaxial dynamic compressive loading regimes, including shear.

Reference	Study design/investigation	Cell source (*n*), scaffold, and preculture duration	Loading parameters	Results [PG = proteoglycans, Eeq = equilibrium, Edyn = dynamic, Ey = Young’s, and H(A) = aggregate]
Waldman et al. (2003)	Uniaxial compression versus shear	Adult bovine carpal-metacarpal chondrocytes on calcium phosphate ceramic surface precultured for 28 days	Frequency: 1 Hz Amplitude: 5% compress or 2% shear Duration: 400 cycles (6 min)/48 h for 28 days Max duration: 1.4 h	RNA: not assessed PG: ↑ only shear load Collagen: trended ↑ for only shear load Biomechanics: ↑ Eeq for both compressive and shear load

Grad et al. (2006)	Effect of unidirectional and multidirectional loading	3- to 4-month-old primary bovine chondrocytes in polyurethane scaffold precultured for 5 days	Frequency: 1 Hz Amplitude: 10% compress ±25° oscillation Duration: 2× 1 h/day for 5 days Max duration: 10 h	RNA: ↔ for dynamic compression only, ↑ in PRG4, ACAN, COMP, COL2, and TIMP3 shear/multiaxial relative to dynamic compression PG: ELISAs: ↔ COMP, PRG4, or HA for compression, ↑ COMP, PRG4, HA for multiaxial relative to dynamic compression Collagen: not assessed Biomechanics: not assessed

Waldman et al. (2007)	Effect of multiaxial loading	6- to 9-month-old primary bovine chondrocytes (*n* = 2–3 pooled) on calcium phosphate ceramic surface precultured for 28 days	Frequency: 0.5 Hz Amplitude: 2 or 5% compress and shear Duration: 400 cycles (6 min)/48 h for 6 days Max duration: 18 min	RNA: not assessed PG: ↑ only for 5% compression + 5% shear Collagen: ↑ only for 5% compression + 5% shear Biomechanics: ↑ in Eeq and G(shear), strain stiffening for compression + shear load

Wernike et al. (2007)	Additive effect of low oxygen environment to multiaxial loading	4- to 8-month-old primary bovine chondrocytes in polyurethane scaffold precultured for 6 days	Frequency: 0.5 Hz Amplitude: 10% compress ±25° oscillation Duration: 1 h/day, 6 days/week for 28 days Max duration: 28 h	RNA: ↓ COL1 in load and low oxygen at days 8 and 34, ↔ COL2, ACAN PG: ↓ with loading, but not compared with control Collagen: not quantified Biomechanics: not assessed

Bian et al. (2010)	Investigated immediate versus delayed loading and addition of shear	2- to 4-year-old expanded canine chondrocytes in 2% agarose hydrogel precultured for 0, 14, or 28 days	Frequency: 1 Hz Amplitude: 10% compress ±180° oscillation Duration: 3 h/day, 5 days/week for 42 days Max duration: 90 h	RNA: not assessed PG: ↔ any loading regime at any time point Collagen: ↔ any loading regime at any time Biomechanics: ↑ Ey continuous load at days 28 and 56, delayed load at day 56. ↑ Edyn all loading regimes at days 56, reduced μ (friction coefficient) for shear load

Shahin and Doran (2011)	Effect of dynamic loading after preculture in perfusion or shaking flasks with varied cell concentration and scaffold	16- to 20-week-old expanded human fetal epiphyseal chondrocytes (*n* = 3 pooled) in poly-glycolic acid (PGA) or PGA + 1.2% alginate scaffold precultured for 3 or 14 days	Frequency: 0.05 Hz Amplitude: 8.7% compress + 3 rpm revolution strain Duration: 10 min/day for 17 days Max duration: 2.8 h	RNA: not assessed PG: ↑ for all scaffold and cell density variations with loading and shaking flask preculture. Highest content with long preculture in perfusion Collagen: ↑ for all scaffold and cell density variations with loading. Highest content with long preculture in perfusion Biomechanics: not assessed

Wang et al. (2013)	Effect of cell expansion and passage on response to loading	3- to 4-month-old primary or expanded bovine chondrocytes (*n* = 3) in polyurethane scaffold precultured for 1 or 14 days	Frequency: 1 Hz Amplitude: 10% compress ±25° oscillation Duration: 10 min/day for 17 days Max duration: 2.8 h	RNA: ↑ COL2, COMP, ACAN, and PRG4 for continuous and delayed load and p0 and p3 chondrocytes PG: ↑ continuous and delayed loading Collagen: ↑ type II, ↔ type I on IHC Biomechanics: not assessed

Pourmohammadali et al. (2013)	Effect of perfusion and multiaxial loading	Primary bovine chondrocytes in 3% agarose hydrogel precultured for 7 days	Frequency: 8–14 mm/s Amplitude: 18% + shear flow Duration: 30 min/day for 21 days Max duration: 10.5 h	RNA: not assessed PG: ↔ perfusion ± compression and shear Collagen: not assessed Biomechanics: not assessed

Hilz et al. (2013)	Effect of low-frequency, low-energy electromagnetic fields combined with multiaxial loading	2- to 3-month-old primary bovine chondrocytes in polyurethane scaffold precultured for 7 days	Frequency: 1 Hz Amplitude: 10% compress ±25° oscillation Duration: 2× 1 h/day every other day for 21 days Max duration: 16 h	RNA: ↑ COL2/COL1 ratio, PRG4, ↔ MMP3, MMP13, COMP, and SOX9 PG: ↑ with load Collagen: ↑ type II, ↓ type I on Remmele score Biomechanics: not assessed

Neumann et al. (2015)	Response of chondroprogenitors to dynamic loading and/or BMP2	30- to 75-year-old expanded clonal human articular cartilage progenitor cells (*n* = 4) in polyurethane scaffold precultured for 3 days	Frequency: 1 Hz Amplitude: 10% compress ±25° oscillation Duration: 1 h/day, 6 days/week for 7 or 28 days Max duration: 24 h	RNA: ↑ COL1 (day 7), ACAN (days 7 and 28),↑ COLX (day 28 with BMP2); COL2 undetectable PG: ↑ with load, ↓ with addition of BMP2 Collagen: not assessed Biomechanics: not assessed

## Conclusion

**Figure 2:**
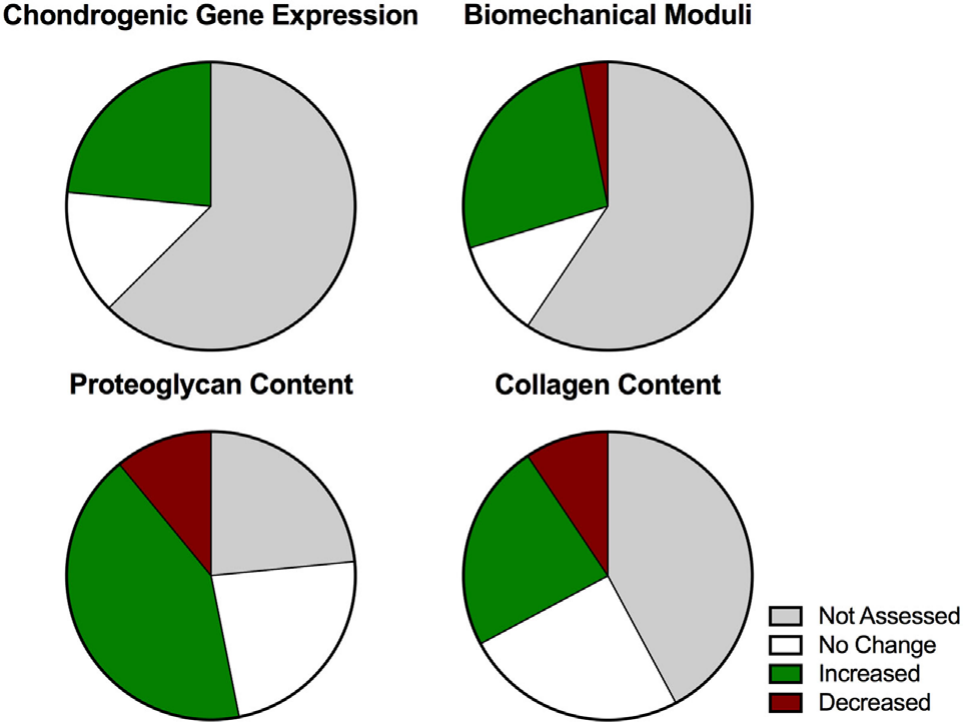
Graphic representation of compiled results (*n* = 63) from studies investigating dynamic compressive stimulation of chondrocytes to summarize the overall effect with respect to standard outcome measures.

Before starting this review, the authors were convinced that dynamic compressive loading of chondrocytes is an important factor to include when creating articular cartilage-like tissue *in vitro*. After reviewing the literature, it has become clear that there exists little standardization in the work done in this area to date, such that the variability of results obtained weakens the basis for our conviction. When plotted by percentages of positive, negative, or no effect data from this collective set of studies, we can clearly see that dynamic loading had a positive effect on the expression of chondrogenic genes in a majority of studies in which they were examined (Figure 2). This is also true for biomechanical moduli and proteoglycan content; although some negative effects were noted for these parameters in some studies, a greater proportion of studies reported positive effects than the sum of “decreased” plus “no change” results. However, this was not true for effects of dynamic compression on collagen content. This is a very important distinction that may be highly relevant for the creation of functional articular cartilage implants with the extracellular matrix and stiffness necessary to fulfill its biomechanical role in the joint. Finding loading parameters that increase collagen production would seem to be a priority going forward. To date, agarose represents the most standardized substrate in which to seed cartilaginous cells for loading studies. Upon review, we noted that the following loading conditions favor the development of an articular cartilage-like tissue phenotype in this type of hydrogel: dynamic compressive loading at physiologic frequency (1 Hz); delayed loading after a preculture period to allow initial extracellular matrix elaboration; intermittent loading regimes with substantial daily rest periods; and total loading duration greater than 50 h.

Further, adequate characterization of cells with respect to phenotype based on donor age, primary versus expanded populations, and site of derivation (superficial versus deep, load bearing versus non-load bearing) is necessary to fully appreciate the response of cells to a complex loading regime. Primary cells retain their chondrocytic phenotype. However, if expansion of cells is needed, techniques that effectively limit the dedifferentiation seen in monolayer will need to be further developed. Expansion at physioxia, or with certain growth factors have been shown to influence phenotypic drift, but no such techniques were used in the studies we analyzed (Jakob et al., 2001; Mandl et al., 2004; Schrobback et al., 2012). While focus on substrate, cells, and loading environment is necessary to define tissue engineering within a dynamic loading environment, many studies failed to appreciate or report the added effects of soluble factors, which may independently influence cellular metabolism and tissue development. All of these considerations highlight the complexity of tissue engineering within a bioreactor, and all elements must be given adequate consideration to draw meaningful conclusions.

Those conclusions notwithstanding, it is clear from our analysis that it is time for standardized protocols and analyses to be introduced. At the gene level, Jeon et al. (2012, 2013) showed that gene expression is dependent on the time of RNA extraction relative to loading; thus, studies that report gene expression without standardization or consideration of RNA extraction timing, potentially report results out of context. As with every story told regarding tissues with extensive extracellular matrices, it is vital that protein-level analysis accompany gene analysis, something not always considered appropriately in chondrocyte dynamic loading studies. Gene expression does not necessarily correlate to structural proteins being secreted and incorporated into the extracellular matrix of developing tissues. Just as gene expression helps to define cellular phenotype, so too is collagen protein quantification and differential characterization of collagen type by quantitative methods such as ELISA necessary to define the tissue phenotype of cartilaginous tissues. Furthermore, paying close attention to the differences in bioreactor design, loading regime, biomaterial, cell source, and culture conditions between studies is vital when drawing conclusions about their relative significance. While this review exclusively includes dynamic loading effects on chondrocytes, the conclusions drawn apply equally to studies with other cell types, namely stem/progenitor cells, also used for cartilage tissue engineering. At the very least, the field would greatly benefit from transparency in reporting sample size, including biological versus technical replicates, which were neglected in greater than half of studies evaluated. The galvanizing effects of adding shear to dynamic compression protocols provides a very important lesson regarding the “deconstruction of complexity” that is present in *in vitro* loading systems: care must be taken when drawing conclusions from the results obtained in such simplified conditions in the laboratory.

This review focused exclusively on *in vitro* methods to generate articular cartilage utilizing chondrocytes cultured in a dynamic mechanical environment. These various methods have been developed with the goal to generate articular cartilage for the repair of focal tissue defects following traumatic injury. For successful translation of these methods to clinical application consideration must also be given to scalability, reproducibility, and cost. Presently, there are no FDA approved biologic implants grown in a dynamic compressive bioreactor; the first therapeutic would require substantial investment for comparison with either microfracture or autologous chondrocyte implantation for repair of focal articular cartilage defects. Furthermore, the few studies utilizing human-derived cells in dynamic compressive bioreactors did not reach significance for most outcome parameters measured, highlighting wide variation between donors. It must also be noted that these studies primarily utilized chondrocytes derived from osteoarthritic tissue, which is not representative of either the target tissue or the intended population for focal cartilage repair. Moving forward, we must learn from these studies not only to work toward standardization of bioreactors but also to pay attention to translation for clinical application with use of human cells from non-diseased tissue.

## Ethics Statement

No human or animal subjects were used in this study.

## Author Contributions

DA and BJ conceived of the review topic and participated in manuscript writing, editing, and preparation for publication. DA reviewed the primary literature and cataloged the results.

## Conflict of Interest Statement

The authors declare that the research was conducted in the absence of any commercial or financial relationships that could be construed as a potential conflict of interest.
